# Editorial: New therapeutic targets for human placental angiogenesis disease

**DOI:** 10.3389/fphar.2014.00263

**Published:** 2014-12-01

**Authors:** Carlos A. Escudero

**Affiliations:** Vascular Physiology Laboratory, Group of Investigation in Tumor Angiogenesis (GIANT), Group of Research and Innovation in Vascular Health (GRIVAS Health), Department of Basic Sciences, University of Bío-BíoChillán, Chile

**Keywords:** placenta, angiogenesis, therapy, hypothesis, review

Development of placental vascular tree is structurally and functionally required for both adequate placental growth and delivery of nutrients from mother to the fetus. Impaired placental angiogenesis has been implicated in the pathophysiology of pregnancy complications which have immediate and long-lasting effects on the mother and her child; such complications include fetal growth restriction and macrosomia as well as pre-eclampsia and gestational diabetes. The mechanisms underlying the deregulation of placental angiogenesis include a misbalance between the secretion and activity of pro-angiogenic vs. anti-angiogenic factors. Despite this, therapies for improving placental angiogenesis in pathological pregnancies have not been directly tested in humans and the aim of this Research Topic in Frontiers is to highlight potential therapeutic targets.

Physical activity during pregnancy might be effective for reducing the risk of developing pregnancy complications. In this regard, Rodriguez and Gonzalez (2014) explain how physical activity affects placental endothelial shear stress and vasodilation, via synthesis and release of nitric oxide (NO). Whilst these processes are relatively well-described in the adult circulation, they are not completely understood in the feto-placental circulation. Then, they analyze how training affects hemodynamics in the mother, which may favor blood supply to the placenta, favoring placental angiogenesis, glucose and oxygen delivery and thereby fetal growth and development. Considering this concept, the potential beneficial effects of moderate levels of maternal exercise may constitute a non-pharmacological intervention for improving maternal and fetal hemodynamic alterations observed during pathological pregnancies such as pre-eclampsia, gestational diabetes, or intrauterine growth restriction.

On the other hand, Herrera et al. (2014) characterizes the response of the umbilical-placental vasculature to hypoxia induced experimentally or through living at high-altitude. They later describe how hypoxia and oxidative stress may impact placental establishment and therefore have consequences for embryonic development from the early stages of gestation. The authors also highlight the implications of a prolonged hypoxic environment in inducing adaptive responses of the placenta in pregnancies at high altitude, or conversely in the development of placental vascular pathologies such as those observed during intrauterine growth restriction and pre-eclampsia. The clinical significance of these findings are discussed, emphasizing that balancing levels of oxidative stress may be a target for improving placental vascular alterations.

Impaired remodeling of maternal spiral arteries by invasive placental trophoblast is thought to be the primary cause of intrauterine hypoxia and the pathophysiology of pre-eclampsia and/or intrauterine growth restriction. In this regard, Salomon et al. (2014) presented novel data which improve our understanding in the interactions of trophoblast with vascular smooth muscle cells (VSMC) via exosomes. They assess exosomes release and content in two extravillous trophoblast cell lines (JEC-3 and HTR-8/SVneo), and relate their observations with the capacity of these exosomes for promoting migration of VSMC. They found high release, differential composition, and high promotion of VSMC migration in exosomes released from HTR-8/SVneo cells compared with JEC-3 cells. Interestingly, their findings indicate that modulation of VSMC migration depends on exosome cargo, exosomal structural integrity, and intracellular incorporation of these exosomes into VSMC. These promising results highlight the potential of exosomes as diagnostic biomarkers of normal or abnormal placentation, or perhaps may constitute an alternative method for introducing molecules with a therapeutic aim.

Bidwell and George (2014), also show that employing a carrier protein called elastin-like polypeptide (ELP) may provide a method for delivering therapeutic agents/drugs to the placenta during pathological pregnancies, including pre-eclampsia. These particular peptides may offer many advantages since researchers could manipulate their length, sequence, and therefore biochemical properties in order to selectively target a particular cell and/or tissue. More importantly, due to the high molecular weight of ELP, it cannot cross the placenta avoiding fetal exposure and potential developmental defects. Preclinical studies are currently underway exploring whether fusing ELP with proteins like vascular endothelial growth factor (ELP-VEGF), the p50 subunit of NF-kB or with the Nox2 docking sequence (Nox2ds) affects their half-life, or activity.

Cindrova-Davies (2014) provides an overview of the pathophysiology of pre-eclampsia. In particular, the contribution of hypoxia, the equilibrium between oxidants-antioxidants, soluble vascular endothelial growth factor receptor 1 (s-Flt1), the bioavailability of NO and hydrogen sulfide (H_2_S) and the levels of pro-inflammatory and endoplasmic reticulum (ER) stress in the development of this disease are discussed. Considering this biological background, She identifies potential pharmacological targets for improving placental function in pre-eclampsia. These pharmacological tools may include ER chaperones such as ursodeoxycholic acid, as well as vasoactive molecules including L-arginine, NO-donors, H_2_S and statins; some of them are indeed being tested in clinical trials.

Also on the topic of pre-eclampsia, Escudero et al. (2014) propose a challenging hypothesis that the impaired adenosine-mediated placental angiogenesis observed in pre-eclampsia might also be present in the offspring at birth and lead to a reduction in microvascular formation and compromised hemodynamic regulation. According with this hypothesis, adenosine could constitute another avenue for recovering both impaired placental angiogenesis and future complication in the offspring.

On the other hand, Guzman-Gutierrez et al. (2014) raise the notion that the placenta controls the bioavailability of thyroxine (T_4_) and tri-iodothyronine (T_3_) in the fetal circulation. They propose that the placenta may respond in an adaptive fashion to low maternal T_4_ although placental control of T_3_/T_4_ may be impaired during chronic maternal hypothyroxemia or hypothyroidism. They also present evidence suggesting that T_3_/T_4_ controls endothelial function, via effects on placental angiogenesis and the synthesis and release of vasodilators and vasoconstrictors. Additionally, they suggest that low levels of T_4_ observed during gestational diabetes might contribute to impaired vascular function observed in this disease.

Nevertheless, Saez et al. (2014) review how ER stress could impair endothelium migration, one of the initial steps in the angiogenesis process. In addition they suggest that obesity, a well-described condition associated with ER stress, could also drive impaired placental angiogenesis. Their analysis includes characterizing potential intracellular signaling pathways that could link obesity mediated ER stress with alteration in the pro-migratory signals. Then, as described also by Cindrova-Davies in this issue, ER stress modulators might constitute a potential therapy for improving placental angiogenesis.

For understanding regulators of angiogenesis process, Murthi et al. (2014) describe how homeobox genes regulate the transcription of genes essential for angiogenesis in the human placenta. Their analysis includes a description of homeobox genes differentially expressed in the macrovascular and microvascular endothelium derived from the feto-placental vessels, which may improve our understanding of physiological and pathological placental angiogenesis. Therefore, manipulating the expression of homeobox genes and/or their targets in the placenta may serve as an alternate method to improve the outcome of pregnancies compromised by perturbed placental angiogenesis.

Another, cutting-edge analysis related with differential gene expression in placental endothelium and the vascular tree is reviewed by Casanello et al. (2014). The authors focus on how epigenetic mechanisms play a role in endothelial physiology, providing the example that the promoters of endothelial nitric oxide synthase (eNOS), and arginase 2 (Arg-2) are differentially methylated in the endothelium of arteries or veins, as well as in macro or microcirculation in the human feto-placental vasculature. Interestingly, by studying the patterns of DNA methylation, they suggest an “arterization” of human chorionic endothelium vein derived from pregnancies with intrauterine growth restriction. They also suggest that changes in the activity and/or expression DNA-methytransferases might comprise potential new targets for both understanding the control of gene expression in the fetal-placental unit, as well as for identifying new potential targets for therapy.

As presented in this Research Topic, the study of placental angiogenesis constitutes a niche of research not only for understanding pathophysiology of human pregnancy diseases such as pre-eclampsia, intrauterine growth restriction, or gestational diabetes; but also for the development of therapeutic tools which promote placental vascularization and function and thus improve fetal development with lasting effects into adult life. As described in each paper in this Research Topic so much is unknown in this field, therefore I would like to encourage researchers to continue contributing to our understanding of placental angiogenesis during normal and pathological conditions. Finally, but more importantly, I would like to thank all authors who have contributed papers, as well as, the reviewers and editorial board for helping us in underscore the importance of this Research Topic.

## Conflict of interest statement

The author declares that the research was conducted in the absence of any commercial or financial relationships that could be construed as a potential conflict of interest.
